# The Hospital World

**Published:** 1911-06-17

**Authors:** 


### The Hospital World.

### The Royal Free Hospital's New Secretary.

As we go to press we learn that Mr. Reginald R. Garratt has been appointed secretary to the Royal Free Hospital in succession to Mr. Conrad Thies, who is resigning this post. Mr. Garratt, we understand, was secretary to the Women's Jubilee Memorial Fund to Queen Victoria and to the Infants' Hospital during the first three years of its existence.

### Prize Giving by Lady Minto at the School of Medicine for Women.

Lady Minto distributed last week the prizes and certificates at the London School of Medicine for Women attached to the Royal Free Hospital. Mrs. Garrett Anderson, M.D., the president of the school, who occupied the chair, said that the school had been getting on well as regarded numbers, and that the entries were larger than in the preceding year. Lady Minto then said that for some years medical and nursing work had been a source of considerable interest to her, and she had had great opportunities of judging the progress of medical efficiency, especially in Canada and India. As president for the past five years of the National Association for Providing Medical Aid for the Women of India, known, till lately, as the Countess of Dufferin's Fund, she had been impressed with the splendid work which had been accomplished, especially for those who were behind the veil, by the introduction of Western science into the hospitals of the East. She could not speak too highly of the way in which medical women, many of whom had been trained within the walls of that admirable school at the Royal Free Hospital, had succeeded in their most difficult and arduous work in the trying climate of India. There was much scope for work, but the pay and position of medical women in India were not altogether satisfactory at present. She trusted that an efficient service would be formed in the near future with the full support and sympathy of the Indian Government.

### A Practitioner's Gift to the Public.

A well-known Reading practitioner, Dr. J. Boyd Hurry, J.P., has been instrumental in presenting a beautiful estate to the public. One Tree Hill, Sevenoaks, Kent, was presented to the National Trust by Mrs. and Dr. Hurry for the benefit of the nation, and on last Saturday was dedicated to the public and declared open. Dr. Hurry, as the author of papers and a recently published work on "Vicious Circles," is to be congratulated on his beneficent action, by which he has broken up what would in all probability have given rise in the future to yet another vicious circle in the hands of the builder.

### Sir Thomas De La Rue's Will.

The enormous fortune of Sir Thomas Andros De la Rue, the gross value of whose estate has now been declared in round figures at £822,000, does not yield many bequests to hospitals. The chief charitable beneficiary under it is the Royal Hospital for Diseases of the Chest, in City Road, which will receive £1,000, a legacy peculiarly apt in view of the new shelter and open air accommodation now in contemplation for the hospital's grounds, while St. George's Hospital and the London Fever Hospital will benefit by legacies of £250 each. Sir Thomas' father was a doctor, not of medicine, but of science, and the said fortune, as everyone knows, has been made out of that special and privileged department of printing which is concerned with the manufacture of postage-stamps and playing cards. It is to be noted that the will, in which figure only three charities, all of which are hospitals, contains many legacies to servants and employees.

